# Genomic and Functional Characteristics of Human Cytomegalovirus Revealed by Next-Generation Sequencing

**DOI:** 10.3390/v6031049

**Published:** 2014-03-05

**Authors:** Steven Sijmons, Marc Van Ranst, Piet Maes

**Affiliations:** Laboratory of Clinical Virology, Rega Institute for Medical Research, K.U.Leuven, Minderbroedersstraat 10, Leuven BE-3000, Belgium; E-Mails: marc.vanranst@uzleuven.be (M.V.R.); piet.maes@rega.kuleuven.be (P.M.)

**Keywords:** cytomegalovirus, genomics, transcriptomics, next-generation sequencing, drug resistance, deep sequencing

## Abstract

The complete genome of human cytomegalovirus (HCMV) was elucidated almost 25 years ago using a traditional cloning and Sanger sequencing approach. Analysis of the genetic content of additional laboratory and clinical isolates has lead to a better, albeit still incomplete, definition of the coding potential and diversity of wild-type HCMV strains. The introduction of a new generation of massively parallel sequencing technologies, collectively called next-generation sequencing, has profoundly increased the throughput and resolution of the genomics field. These increased possibilities are already leading to a better understanding of the circulating diversity of HCMV clinical isolates. The higher resolution of next-generation sequencing provides new opportunities in the study of intrahost viral population structures. Furthermore, deep sequencing enables novel diagnostic applications for sensitive drug resistance mutation detection. RNA-seq applications have changed the picture of the HCMV transcriptome, which resulted in proof of a vast amount of splicing events and alternative transcripts. This review discusses the application of next-generation sequencing technologies, which has provided a clearer picture of the intricate nature of the HCMV genome. The continuing development and application of novel sequencing technologies will further augment our understanding of this ubiquitous, but elusive, herpesvirus.

## 1. Introduction

**Figure 1:**
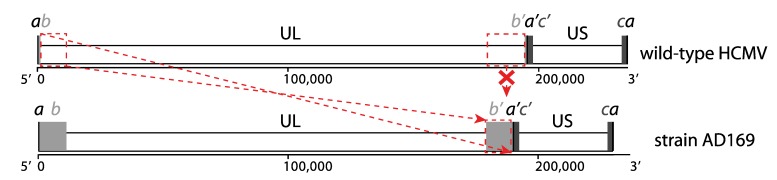
Class E genome of HCMV. The unique long and unique short regions are indicated as UL and US. Repeat regions are indicated as *a*, *b* and *c* sequences, where primes designate inverted orientations. Sequences *ab* and *b’a’* correspond to the terminal/internal repeat long (TRL/IRL); sequences *a’c’* and *ca* correspond to the internal/terminal repeat short (IRS/TRS). Top: typical genome arrangement of wild-type strains; bottom: genome arrangement of strain AD169 is given as an example of a laboratory-adapted strain. Genome rearrangements (deletion of UL 3’ end and replacement by an inverted copy of UL 5’ end) that have occurred during extensive passaging are indicated in red between the wild-type and laboratory-adapted configurations.

Human cytomegalovirus (HCMV), the prototype member of the herpesvirus subfamily *Betaherpesvirinae*, has a worldwide distribution and infections with this virus are extremely common. Seroprevalences in the adult population vary from 45% to 100%, increasing with age and varying with geographic location and socio-economic background [1]. HCMV causes only mild to no symptoms in immunocompetent individuals, but the virus is never cleared and establishes a latent infection for the lifetime of its host [2]. Primary infection, reactivation, or reinfection of immunocompromised individuals, such as transplant recipients and AIDS patients, results in considerable morbidity and mortality [3]. Furthermore, it is the most important congenital infection in both developed and developing countries, causing sensorineural hearing loss and neurodevelopmental delays [4]. The double‑stranded DNA (dsDNA) genome of wild-type HCMV strains has a size of around 235 kb, which is longer than all other human herpesviruses and one of the longest genomes of all human viruses in general. It has the characteristic herpesvirus class E genome architecture, consisting of two unique regions (unique long UL and unique short US), both flanked by a pair of inverted repeats (terminal/internal repeat long TRL/IRL and internal/terminal repeat short IRS/TRS). Both sets of repeats share a region of a few hundred bps, the so-called “*a* sequence”; the other regions of the repeats are sometimes referred to as “*b* sequence” and “*c* sequence” (Figure 1). The genome exists as an equimolar mixture of four genomic isomers by inversion of UL and US regions [5].

The commercial introduction of new DNA sequencing technologies, referred to as next-generation sequencing (NGS), has immensely impacted the field of genomics. These novel technologies generate a massive throughput at a considerably lower per base cost than traditional Sanger sequencing, and obviate the need for laborious cloning procedures [6,7,8]. NGS has already shown its promise in virological research with applications in virus discovery and metagenomics, whole genome analysis, deep sequencing of viral populations, novel diagnostic assays, and studies of virus-host interactions through transcriptome and epigenome studies [9,10,11]. In this review, we will highlight the recent progress that has contributed to the understanding of HCMV genomics through the application of NGS technologies.

## 2. HCMV Genomics before the Introduction of NGS

### 2.1. Genome Alterations during Cell Culture Adaptation

When the first complete genome sequence of HCMV was published in 1990, it was the largest contiguous sequence generated at the time [12,13]. The authors estimated that the effort to sequence the genome of the laboratory-adapted strain AD169 with M13 shotgun cloning and Sanger sequencing was equivalent to a 12-year workload for one person. The laboratory-adapted strains AD169 and Towne had been passaged extensively in human fibroblast cell lines and were found to cause no or very low virulence in seronegative individuals during vaccine studies [14,15,16]. In contrast, the Toledo strain had been passaged significantly less and produced mononucleosis syndromes when administered to seropositive individuals [17]. When genome regions of AD169, Towne, and Toledo were compared through restriction enzyme profiles, hybridization, and sequencing techniques, it was observed that AD169 and Towne had lost genome segments of 15 kb and 13 kb, respectively. These segments were situated at the 3’ end of the UL region (sometimes referred to as the UL/*b*’ region) and were replaced by an inverted copy of the 5’ end of the UL region, leading to an enlargement of the *b* repeats (Figure 1). This missing UL/*b*’ segment was also found to be present in an additional set of five low-passage clinical isolates like Toledo, confirming that it is a universal hallmark of wild-type HCMV strains and clearly contains factors that are dispensable for fibroblast replication, but essential for virulence *in vivo* [18,19].

**Figure 2:**
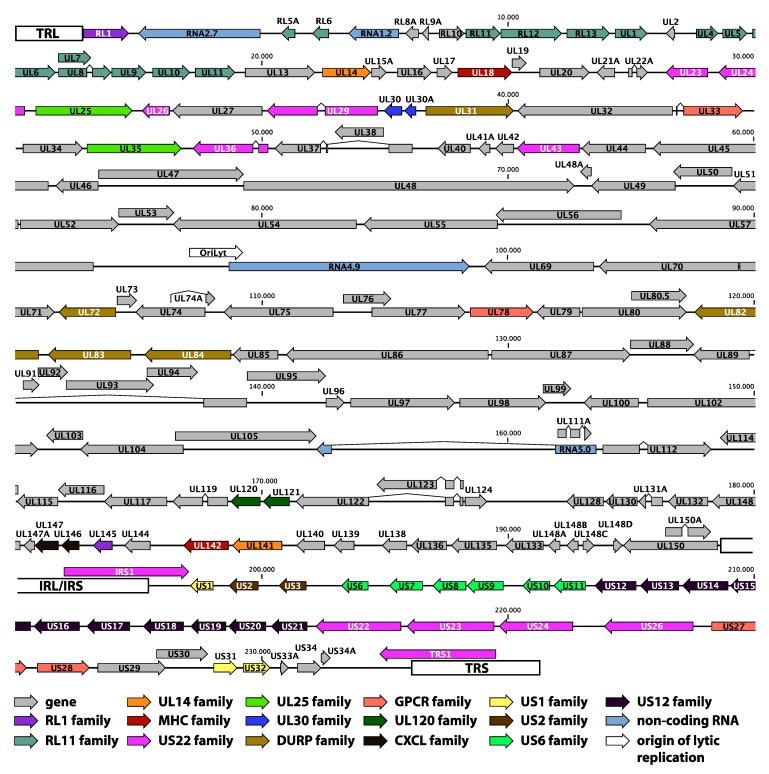
Genome annotation of the low-passage HCMV strain Merlin (GenBank accession NC_006273). The dsDNA genome is visualized as a single line; nucleotide positions are given in bps. Terminal and internal repeat regions (TRL, IRL/IRS and TRS) are indicated with white boxes. Arrows represent genes; different gene families are designated with different color codes, as illustrated below the genome. The four large non-coding RNAs and the origin of lytic replication are also represented.

In addition to these genome rearrangements, strains AD169 and Towne display more subtle alterations of their coding capacity in genes inside—variants of AD169 and Towne with a more or less intact UL/*b*’ region exist [20]—and outside the UL/*b*’ region. AD169 has open reading frame (ORF) disrupting mutations in genes RL5A, RL13, UL36, and UL131A; Towne is affected in genes RL13, UL1, UL40, UL130, US1, and US9 [20,21,22,23,24]. Even the low-passage strain Toledo is mutated in genes RL13, UL9 and UL128 [25]. Almost all strains passaged in fibroblast cell cultures display one or more mutations in the UL128 locus (UL128L) and in the genes of the RL11 family, indicating a role in cell tropism for these gene products. UL128L consists of genes UL128, UL130, and UL131A (Figure 2). and its products form a complex with the viral glycoproteins gH and gL. While this complex is dispensable for growth in fibroblasts, it is essential for endothelial and epithelial cell tropism [26,27,28]. The RL11 gene family contains 14 genes at the 5’ end of the UL region (RL5A, RL6, RL11-UL1, UL4-UL11, Figure 2) that are dispensable for growth in fibroblasts and are functionally poorly characterized [23,29,30]. Several of these genes show a remarkable genetic variability between different clinical isolates [25,31]. The majority of the RL11 genes have a characteristic domain (RL11D) that shares homology with the CR1 domain of the adenovirus E3 genes [23]. These proteins could function as modulators of a set of variable host proteins, and similarities to the immunoglobulin IgD family have been proposed [12]. Recently, some RL11 genes have indeed been implicated in immune evasive functions [32,33,34].

While the consensus sequence of the low-passage strain Merlin only has a mutation in gene UL128, cloning of the strain into a bacterial artificial chromosome (BAC) vector showed that it was also defective in gene RL13 [25,35]. Since RL13 mutations were present at different sites in different clones, this was not noticeable in the consensus sequence. Repair of UL128 in the Merlin BAC repressed replication in fibroblast cells, but not in epithelial cells and, consequently, novel mutations only emerged in fibroblasts. On the contrary, repair of RL13 impaired replication in fibroblast cells as well as in epithelial cells, and mutants appeared rapidly in both cell types. Mutational dynamics of clinical HCMV isolates were further analyzed in fibroblasts, epithelial, and endothelial cells by recording all mutations in four isolates up to passage 50 or more [36]. Several additional genes were mutated in some strains, but only UL128L and RL13 invariably mutated in fibroblast cells (RL13 in all cell types). Taken together, these studies stress the inherent instability of HCMV isolates when passaged in cell culture. Thus, *in vitro* studies with HCMV strains should allow for these limitations, and researchers should be mindful of the genetic changes that have altered the coding capacity and functionality of the strain under study.

### 2.2. Genome Annotation

The publication of the first complete genome sequence of HCMV was accompanied by a first genome annotation, predicting 208 ORFs that were potentially protein encoding [12]. Only ORFs encoding for proteins with a minimum length of 100 amino acids were considered, with a maximal overlap of 60% between ORFs. As the authors expected, this preliminary annotation wrongly predicted some ORFs and at the same time excluded genuine ORFs that were either too small or highly spliced. Since the results were based on the laboratory-adapted strain AD169, the ORFs encoded by the UL/*b*’ region (Figure 1) were also missed [18,19]. The annotation was further refined by comparison with chimpanzee, rhesus and murine cytomegalovirus genomes [37,38] and by sequence analysis of additional clinical isolates of HCMV [25,39]. The low-passage strain Merlin has become the reference strain for wild-type HCMV, and its sequence entry (NCBI GenBank accession NC_006273, Table 1) currently contains a set of 170 genes. Several sets of genes share some sequence similarity and are thought to have originated from duplication events and subsequent divergence of structure and function [12,13]. These 15 gene families are indicated on the genome map in Figure 2.

**Table 1:** HCMV complete genome sequences available on NCBI GenBank, listed in order of submission date.

GenBank accession	Strain name	Clinical source	Passage history	Ref.	Submission date
**X17403**	AD169	Adenoids of a 7-year old girl	Passaged extensively in human fibroblasts	[12]	December 6, 1989
**BK00039’**	AD169 varUK	Adenoids of a 7-year old girl	Passaged extensively in human fibroblasts	[40]	May 1, 2002
NC_006273*GU179001	Merlin	Urine from a congenitally infected infant	Passaged 3 times in human fibroblasts	[25]	September 27, 2002
**AY315197**	Towne varS	Urine of a 2-month-old infant with microcephaly and hepatosplenomegaly	Passaged extensively in human fibroblasts	[30]	June 6, 2003
**AC146851**	Towne-BAC	Urine of a 2-month-old infant with microcephaly and hepatosplenomegaly	BAC clone from a plaque purified Towne derivative (varS)	[39]	October 14, 2003
**AC146904**	PH-BAC	Transplant patient with HCMV disease	BAC clone from isolate PH (passaged less than 12 times)	[39]	October 21, 2003
**AC146905**	Toledo-BAC	Urine from a congenitally infected infant	BAC clone from a plaque purified Toledo derivative	[39]	October 21, 2013
**AC146906**	TR-BAC	AIDS patient with CMV retinitis	BAC clone from isolate TR	[39]	October 21, 2013
**AC146907**	FIX-BAC	Cervical secretions of a pregnant woman with a primary HCMV infection	BAC clone from isolate VR1814	[39]	October 21, 2013
**AC146999**	AD169-BAC	Adenoids of a 7-year old girl	BAC clone from a plaque purified AD169 derivative (varATCC)	[39]	October 31, 2013
**EF999921**	TB40/E clone TB40-BAC4	Throat wash of a bone marrow transplant recipient	BAC clone from TB40/E passaged 5 times in human fibroblasts and 22 times in human endothelial cells	[41]	June 25, 2007
**FJ527563**	AD169 varUC	Adenoids of a 7-year old girl	Passaged extensively in human fibroblasts	[20]	December 1, 2008
**FJ616285**	Towne varL	Urine of a 2-month-old infant with microcephaly and hepatosplenomegaly	Passaged extensively in human fibroblasts	[20]	January 9, 2009
**GQ221973**	HAN13	Bronchoalveolar lavage	Passaged 3 times in human fibroblasts	[42]	May 28, 2009
**GQ221974**	3157	Urine from a congenitally infected infant	Passaged 3 times in human fibroblasts	[42]	May 28, 2009
**GQ221975**	JP	Post mortem prostate tissue from an AIDS patient	Unpassaged	[42]	May 28, 2009
**GQ396662**	HAN38	Bronchoalveolar lavage	Passaged 2 times in human fibroblasts	[42]	July 17, 2009
**GQ396663**	HAN20	Bronchoalveolar lavage	Passaged 2 times in human fibroblasts	[42]	July 17, 2009
**GQ466044**	3301	Urine from a congenitally infected infant	Unpassaged	[42]	August 7, 2009
**GU179288**	U8	Urine from a congenitally infected infant	Unpassaged	[42]	November 5, 2009
**GU179289**	VR1814	Cervical secretions of a pregnant woman with a primary HCMV infection	Unpassaged	[42]	November 5, 2009
**GU179290**	U11	Urine from a congenitally infected infant	Unpassaged	[42]	November 5, 2009
**GU179291**	AF1	Amniotic fluid	Unpassaged	[42]	November 5, 2009
**GU937742**	Toledo	Urine from a congenitally infected infant	Passaged several times in human fibroblasts	[25]	February 26, 2010
**HQ380895**	JHC	Blood from a bone marrow transplant patient	Plaque purified and passaged 3 times in human fibroblasts	[43]	October 7, 2010
**JX512197**	6397	Urine from a congenitally infected infant	Passaged 3 times in human fibroblasts	-	August 21, 2012
**JX512198**	Davis	Liver biopsy from a congenitally infected infant	Passaged many times in human fibroblasts	-	August 21, 2012
**JX512199**	HAN1	Bronchoalveolar lavage	No information	-	August 21, 2012
**JX512200**	HAN2	Bronchoalveolar lavage	Passaged 3 times in human fibroblasts	-	August 21, 2012
**JX512201**	HAN3	Bronchoalveolar lavage	Passaged 3 times in human fibroblasts	-	August 21, 2012
**JX512202**	HAN8	Bronchoalveolar lavage	Passaged 3 times in human fibroblasts	-	August 21, 2012
**JX512203**	HAN12	Bronchoalveolar lavage	Passaged 3 times in human fibroblasts	-	August 21, 2012
**JX512204**	HAN16	Urine from an infant	Passaged 2 times in human fibroblasts	-	August 21, 2012
**JX512205**	HAN19	Bronchoalveolar lavage	Passaged 2 times in human fibroblasts	-	August 21, 2012
**JX512206**	HAN22	Bronchoalveolar lavage	Passaged 2 times in human fibroblasts	-	August 21, 2012
**JX512207**	HAN28	Bronchoalveolar lavage	Passaged 3 times in human fibroblasts	-	August 21, 2012
**JX512208**	HAN31	Bronchoalveolar lavage	Passaged 2 times in human fibroblasts	-	August 21, 2012
**KC519319**	BE/9/2010	Urine from an infant	Passaged 2 times in human fibroblasts	-	January 23, 2013
**KC519320**	BE/10/2010	Urine from a congenitally infected infant	Passaged 2 times in human fibroblasts	-	January 23, 2013
**KC519321**	BE/11/2010	Urine from an infant	Passaged 2 times in human fibroblasts	-	January 23, 2013
**KC519322**	BE/21/2010	Urine from a pulmonary transplant recipient	Unpassaged	-	January 23, 2013
**KC519323**	BE/27/2010	Urine from a renal transplant recipient	Passaged 4 times in human fibroblasts	-	January 23, 2013
**KF021605**	TR	Vitreous humor from eye of HIV-positive male	Passaged several times in human fibroblasts	[44]	May 9, 2013
**KF297339**	TB40/E clone Lisa	Throat wash of a bone marrow transplant recipient	Generated on human fibroblasts by passaging strain TB40/E once, plaque purifying 3 times and passaging once more	[45]	June 26, 2013

°NCBI GenBank release file 199.0; search performed on 15 January 2014; patent sequences, transgenic strains, and incomplete sequences were not included; *NCBI Reference Sequence (RefSeq); “Update from AD169 entry X17403 by the addition of the 929 bp missing region, encompassing UL42 and UL43, and the correction of sequencing errors.

### 2.3. Genetic Diversity

Complete sequence analysis of several clinical isolates not only assisted the refinement of the genome annotation, but also led to the understanding that several regions of the HCMV genome are variable between different isolates [25,39]. Studies of individual gene sequences from viral glycoprotein genes [46,47,48], virulence-determining genes from the UL/*b*’ region [49,50,51], and RL11 genes [31,52,53] have been conducted to establish the existence of separate clusters of polymorphisms or genotypes (reviewed in [54,55,56]). Despite their variability, individual genotypes display remarkable sequence stability both within the host as in the population, and most genotypes seem to have a worldwide distribution [50,57,58,59,60]. Based on these data, it is hypothesized that the selective forces that have shaped the currently circulating genotypes were active during the evolution of early humans or even earlier and were modulated by founder and bottleneck events. In more recent times, migrations of human populations have redistributed and mixed these genotypes on a worldwide scale [56,59,61]. Furthermore, the very low incidence of gene linkage in the HCMV genome probably illustrates the predominant role of recombination in the generation of the existing genetic diversity [31,50,62,63,64].

The existence of distinct genotypes of several genes has attracted interest because of the potential differences in pathogenicity. If such correlations would be observed, this could provide novel diagnostic tools to tailor medical interventions. While some studies investigating genes UL55 (glycoprotein B) [65,66], UL73 (glycoprotein N) [67,68], and UL144 (TNF-α-like receptor) [69,70,71] have presented data involving specific genotypes with different disease outcomes, there is no overall consensus on these correlations yet (reviewed in [54,55,56]). To establish the feasibility of using viral genotype data as prognostic markers in patient follow-up, more comprehensive studies that include larger sets of variable genes, if not complete genome sequences, will be necessary. In this regard, a study making use of gene sequences from only four genes (UL144, UL146, UL147 and US28) could train an artificial neural network to correctly predict congenitally-infected infants to be symptomatic or asymptomatic at birth in 90% of cases [72]. Considering the progress in sequencing technology and its implementation in HCMV genomics, this type of investigation should now become more feasible on a full genome scale.

## 3. Characterization of Complete HCMV Genomes Using NGS

Table 1 gives an overview of all complete HCMV genome sequences that are currently publicly available. If each individual strain is only counted once—some strains have multiple isolate sequences published—, this amounts to a total of 35 strains; 31 of these strains can be considered low-passage (or unpassaged) clinical isolates. While a considerable proportion of these sequences were still deduced using traditional Sanger sequencing, the labor intensity of these approaches precludes routine and high-throughput application of complete genome sequencing. Over the past 5 years, NGS technologies have begun to show their promise in becoming a novel, scalable, cost-effective, and time-efficient way of characterizing HCMV genome diversity.

The first application of NGS to HCMV genomics was published in 2009 and investigated the genome architecture of laboratory-adapted strains AD169 and Towne in detail (Table 2) [20]. While the published sequences of AD169 (varUK and AD169-BAC/varATCC) and Towne (varS/varRIT3) missed the entire UL/*b*’ region (Table 1), it had already been noted that some variants did seem to contain this region [73,74]. For AD169, this variant constituted a separate stock (varUC), but it was unclear whether this was an AD169 variant with an intact UL/*b*’ or a different strain altogether. For Towne, the original stock was a mixture of the varS/varRIT3 variant, that was cloned into a BAC and sequenced [30,39], and an apparently intact varL variant, for which the UL/*b*’ region had been characterized [25]. The exact nature of the AD169 varUC stock and the mixed Towne stock containing both varS and varL was determined by generating sequencing reads with the Genome Analyzer (Illumina) and mapping these onto appropriate reference sequences with or without the UL/*b*’ region. The results showed that AD169 varUC was indeed an AD169 variant with a nearly intact UL/*b*’ region, only missing a 3.2 kb region affecting genes UL144, UL142, UL141, and UL140. Furthermore, the presence of both varS and varL variants in the Towne stock was experimentally confirmed. In a similar fashion, other studies have sequenced specific transgenic BAC clones of strains Merlin and Towne using NGS to characterize genetic changes that have occurred during passaging and cloning of these BACs (Table 2) [35,75,76].

**Table 2:** Overview of studies on the HCMV genome making use of NGS technology, ranked in chronological order.

First author	Title	Journal	NGS technology	Ref.	Publication date
**Bradley *et al.***	High-throughput sequence analysis of variants of human cytomegalovirus strains Towne and AD169.	J. Gen. Virol.	IGA°	[20]	June 24, 2009
**Cunningham *et al.***	Sequences of complete human cytomegalovirus genomes from infected cell cultures and clinical specimens.	J. Gen. Virol.	IGA°	[42]	November 11, 2009
**Görzer *et al.***	Deep sequencing reveals highly complex dynamics of human cytomegalovirus genotypes in transplant patients over time.	J. Virol.	GSF*	[77]	May 12, 2010
**Stanton *et al.***	Reconstruction of the complete human cytomegalovirus genome in a BAC reveals RL13 to be a potent inhibitor of replication.	J. Clin. Invest.	IGA°	[35]	August 2, 2010
**Görzer *et al.***	The impact of PCR-generated recombination on diversity estimation of mixed viral populations by deep sequencing.	J. Virol. Methods	GSF*	[78]	August 4, 2010
**Jung *et al.***	Full genome sequencing and analysis of human cytomegalovirus strain JHC isolated from a Korean patient.	Virus Res.	GSF*	[43]	January 19, 2011
**Renzette *et al.***	Extensive genome-wide variability of human cytomegalovirus in congenitally infected infants.	PLoS Pathog.	IGA°	[79]	May 19, 2011
**James *et al.***	Cyclopropavir inhibits the normal function of the human cytomegalovirus UL97 kinase.	Antimicrob. Agents Chemother.	IGA°	[80]	July 25, 2011
**Stark *et al.***	High-resolution profiling and analysis of viral and host small RNAs during human cytomegalovirus infection.	J. Virol.	IGA°	[81]	October 19, 2011
**Gatherer *et al.***	High-resolution human cytomegalovirus transcriptome.	Proc. Natl. Acad. Sci. U. S. A.	IGA°	[82]	November 22, 2011
**Bhattacharjee *et al.***	Genetic analysis of cytomegalovirus in malignant gliomas.	J. Virol.	IGA°	[83]	April 11, 2012
**Meshesha *et al.***	The microRNA Transcriptome of Human Cytomegalovirus (HCMV).	Open Virol. J.	IGA°	[84]	April 11, 2012
**Stern-Ginossar *et al.***	Decoding human cytomegalovirus.	Science	IGA°, HiSeq^	[85]	November 23, 2012
**Rossetto *et al.***	Cis and trans acting factors involved in human cytomegalovirus experimental and natural latent infection of CD14 (+) monocytes and CD34 (+) cells.	PLoS Pathog.	MiSeq’	[86]	May 23, 2013
**Sahoo *et al.***	Detection of cytomegalovirus drug resistance mutations by next-generation sequencing.	J. Clin. Microbiol.	GSJ”	[87]	August 28, 2013
**Renzette *et al.***	Rapid intrahost evolution of human cytomegalovirus is shaped by demography and positive selection.	PLoS Genet.	IGA°	[88]	September 26, 2013
**Brechtel *et al.***	Complete Genome Sequence of a Cytomegalovirus Towne-BAC (Bacterial Artificial Chromosome) Isolate Maintained in Escherichia coli for 10 Years and Then Serially Passaged in Human Fibroblasts.	Genome Announc.	MiSeq’	[75]	September 26, 2013
**Brechtel *et al.***	Complete Genome Sequence of a UL96 Mutant Cytomegalovirus Towne-BAC (Bacterial Artificial Chromosome) Isolate Passaged in Fibroblasts To Allow Accumulation of Compensatory Mutations.	Genome Announc.	MiSeq’	[76]	October 24, 2013

° Genome Analyzer (Illumina); * 454 GS FLX (Roche); ^ HiSeq (Illumina); ‘ MiSeq (Illumina); “ 454 GS Junior (Roche).

The previous studies made proper use of NGS technology to elucidate the stock composition of laboratory-adapted strains, but, then again, they could employ the existing sequence information to direct the assembly of the millions of sequencing reads that are generated during a typical Illumina run. In order to apply NGS to the genome characterization of novel clinical isolates, this assembly approach needed some adjustment. Because of the sequence variability in substantial regions of the HCMV genome, direct mapping of NGS reads from unknown isolates to existing reference sequences leads to a lack of coverage in these areas, simply because the novel sequences are too divergent from the chosen reference strain. To assemble the sequence information from novel clinical isolates, alternative approaches were devised that start with a *de novo* or reference-independent assembly of sequence reads (Table 2) [42]. The longer sequences formed by *de novo* assembly, the so-called contigs, are scaffolded against a reference sequence to produce a strain-specific reference that can be used for a mapping or reference-dependent assembly like before. The final strain sequence is optimized through manual inspection of the read alignment and correction of misassemblies by iterative mapping and/or PCR sequencing.

A comparative analysis was made of the effectiveness of sequencing complete HCMV genomes from clinical isolates through both Sanger sequencing of overlapping PCR products and NGS analysis of infected cell cultures and unamplified clinical material with the Genome Analyzer [42]. Both approaches were successful, but the PCR and Sanger sequencing method proved to be much more labor-intensive and, by consequence, less amenable to high-throughput application. However, the NGS approach is not specifically directed towards viral DNA and analyzes the total DNA present in an isolate. Whole cell culture extracts are heavily contaminated with cellular DNA and the viral loads in unamplified clinical material can be very low. This was illustrated for strain 3301 (Table 1); only 3% of sequence reads that were collected directly from the sample were of viral origin. While it was possible to reconstruct the complete genome using these 3% of reads, such an approach also precludes any high-throughput prospect. This limitation was recently confirmed when strain BE/21/2010 (Table 1) was amplified using undirected whole genome amplification and only yielded 12% HCMV-specific NGS reads (Sijmons *et al*, unpublished results). This study did realize higher levels of viral DNA (mostly >90%) by combining limited cell culture amplification, nuclease digestion of unencapsidated (cellular) DNA, purification of viral DNA, and whole genome amplification. A series of validation experiments showed that the generated genome sequences did not undergo major alterations during these procedures and were still representative for the strain in the original clinical isolate.

As discussed previously, cell culture passaging leads to disruptive mutations in genes that are inhibitory or non-essential for growth in that cell type. Genes RL13 and UL128L seem to be the first that are affected when passaging a strain in fibroblast cells [35,36]. Interestingly, RL13 and UL128L genes do not show obvious disruptive mutations in most of the clinical isolates analyzed by NGS after limited culturing [42,43]. This suggests that these strains are still in a very early phase of genetic adaptation to fibroblast replication; although it cannot be ruled out that these genes are mutated at different sites in different clones of the population, like in the case of RL13 in strain Merlin [35]. Several observations imply that some ORF-disrupting mutations may be present in the original clinical isolate and are not an artifact of culturing [42]. Most importantly, the sequence characterization of strains JP and BE/21/2010 directly from clinical material (Table 1) has shown disruptive mutations in genes RL5A, UL9, UL111A, and UL150 that are definitely culture-independent. Furthermore, identical indels and point mutations were shared between unrelated isolates, which suggests that these could be derived from a common ancestor circulating in the human population. Finally, the presence of individual mutations in RL5A, UL1, UL9, and UL111A in passaged strains was confirmed by PCR sequencing of the original sample (Sijmons *et al*, unpublished results). Analysis of a larger number of clinical isolates will reveal the complete set of genes that can be disabled in clinical isolates, their occurrence in different patient populations, and potential implications for strain pathogenicity.

## 4. Deep Sequencing of Intrahost HCMV Populations

Accumulating data shows that infections with multiple HCMV strains are no exceptions, neither in immunocompromised nor in immunocompetent hosts (reviewed in [56]). This could probably be the product of both simultaneous and consecutive virus transmission events [89,90]. Multiple infections could result in a higher pathogenic potential because of *trans*-complementation between strains [91]. This prediction is confirmed by data about the effect of strain multiplicity in transplant patients [92,93,94,95]. Because of the large amounts of sequence reads that are generated by NGS technologies, these are ideally suited to characterize the dynamics of mixed viral populations in greater depth (reviewed in [96,97]). The first study to apply this approach to HCMV populations analyzed PCR amplicons of the hypervariable genes UL73, UL74, and UL139 in lung transplant recipients using 454 GS FLX (Roche) technology (Table 2) [77]. Viral populations consisted of mixtures of up to six genotypes, with one or two types accounting for the majority of the population and the other genotypes present at frequencies of 0.1%–10%. When serial samples of patients were compared, the genotype frequencies fluctuated in a seemingly stochastic fashion. The authors speculated these fluctuations could be caused by sporadic and stochastic events that lead to differential reactivation of latent genomes. While the abundance of the individual genotypes changed, their sequences did not, confirming the stability of hypervariable HCMV genes [44,51,53]. In a follow-up study, the authors warned against the formation of artificial recombinants during PCR amplification when interpreting results from amplicon deep sequencing experiments [78].

The previous studies characterized intrahost population diversity by analyzing a set of genes that are highly variable between hosts. However, applying such a deep sequencing approach to a complete genome does not suffer from a selection bias towards certain genome regions and can provide a more comprehensive picture of the diversity and dynamics of viral populations inside the host. Sequencing complete genomes from unamplified clinical material, results in a low proportion of viral NGS reads, which would impair any deep sequencing effort [42]. Cell culture amplification on the other hand would almost certainly alter the composition of viral populations. Therefore, a workflow was devised that characterized complete HCMV genomes using overlapping PCR amplicons [79,83,88]. As an internal control, BAC clones of AD169 and Toledo strains were resequenced to establish a set of quality filtering thresholds that helped distinguishing genuine intrahost variants from PCR and sequencing errors. In a first study, the viral populations of three congenitally infected infants were characterized [79]. Surprisingly, for a dsDNA virus encoding a polymerase with proofreading capacity, estimates of the genetic diversity of these populations were comparable to quasispecies RNA viruses like HIV and dengue virus. Population variants were clustered in two groups. Variants present at high frequencies (≥90%) accounted for 20% of reads, while low-frequency variants (≤10%) represented 73% of reads. This population structure is comparable to the study of UL73, UL74 and UL139 amplicons in lung transplant recipients, which also found one or two variants present at higher frequencies and a set of low-frequency variants (0.1%–10%) [77]. Interestingly, when ORF-specific intrahost diversities were estimated, ORFs encoding glycoproteins or immune-evasive functions showed the lowest intrahost diversity estimates. While they are considered to have the highest interhost diversity, studies focusing on these ORFs may underestimate intrahost diversity. Compared to the results in congenitally infected infants, intrahost diversity estimates were lower in malignant gliomas [83]. A potential explanation for this discrepancy could be the higher levels of replication during congenital infection, which would lead to a higher accumulation of *de novo* mutations. More data from different patient groups is warranted to provide a broader view of the range of intrahost diversity estimates, the mechanisms that shape them, and potential implications for patient health.

To better understand the dynamics of these genome populations, serial urine and plasma isolates were sampled from five infants with a symptomatic HCMV infection at birth [88]. When serial isolates of the same compartment were compared, the majority of SNPs had a similar frequency, and consensus sequences differed only by 0.2% at the nucleotide level, demonstrating the overall stability of the populations. This result is in clear accordance with previous results that have illustrated the stability of HCMV genotypes *in vivo* [50,57,59]. However, comparison of isolates of different compartments (urine and plasma) at the same time point showed that consensus sequences differed by approx. 1%; comparable to the divergence of HCMV strains from different hosts. Subsequently, the observed dynamics were modeled using both demographic variables (population size and structure) and selective pressures. Intercompartment differentiation was shaped by strong bottleneck events and the calculation of bottleneck timing enabled estimation of the timing of infection and compartment colonization. While the effects of positive selection in the same compartment were small, strong evidence of positive selection was found when comparing different compartments. The large differences between viral populations in plasma and urine pose important questions about how representative the secreted virus (urine) is for the virus that circulates (plasma). It is conceivable that other compartments might show other diversification dynamics. However, the total number of patients analyzed in this study is relatively low and the data about intercompartment diversification are only based on one patient. From the presented data, it is unclear whether this patient was infected by a single strain or multiple strains. Rephrasing the issue: are we looking at the differentiation of one single quasispecies cloud or the segregation of multiple quasispecies clouds that could have had inherent differences in their respective cell tropisms? Analysis of the intercompartment diversity in additional patients will be needed to improve the understanding of the dynamics of these virus populations, which obviously could have very important implications for diagnostics, treatment, and vaccine development.

The deep sequencing capabilities of NGS technology show great promise for the sensitive detection of drug resistance mutations. This approach has already proven its use in drug resistance testing for HIV, HCV, and HBV (reviewed in [10]). While Sanger sequencing of UL54 and UL97 PCR amplicons is still the gold standard for detection of drug resistance mutations in HCMV isolates, this method suffers from a lack of sensitivity, often failing when plasma viral loads drop below 1,000 copies/mL and/or mutant frequencies are lower than 10%–20%. Recently, the use of NGS technology in resistance mutation detection was evaluated for the first time for HCMV [87]. Using the benchtop 454 GS Junior system (Roche), the study showed the ability to reproducibly detect resistance mutations at frequencies lower than 20% and at viral loads lower than 1000 copies/mL. This improvement in sensitivity will help studying the abundance, dynamics, and importance of low‑frequency drug-resistant variants. The high throughput of NGS could result in a decrease of the time and cost of resistance detection. In addition, full genome characterization of drug-resistant variants with NGS can potentially lead to the discovery of new resistance mutations in other genome regions.

## 5. NGS in HCMV Transcriptome Studies

The high throughput of NGS provided new opportunities for the field of transcriptomics; the study of the total coding and non-coding RNA that is transcribed in a given cell type [98]. NGS, often referred to as RNA-Seq in this context, has now surpassed microarrays as the method of choice for transcriptomic research, since it is much better at detecting rare variants and does not depend on *a priori* sequence knowledge. Transcriptomic analysis of HCMV in an infected cell can contribute to the genome annotation by revealing complex transcriptional processes that often cannot be predicted based on the genome sequence alone. HCMV transcription is characterized by the presence of multiple transcripts sharing common 5’ or 3’ ends, complex and adaptable splicing patterns, antisense transcription, and transcription of non-coding and miRNAs (reviewed in [99]). Several NGS-based studies have added important insights into these processes.

The first study to use RNA-Seq in HCMV transcriptome research characterized the polyA RNA content of human fetal foreskin fibroblast cells, 72h after infection with the Merlin strain, when virion production is underway [82]. The four large non-coding RNAs that are encoded in the HCMV genome (RNA2.7, RNA1.2, RNA4.9, and RNA5.0; Figure 2) accounted for a staggering 65.1% of viral transcription. These RNAs probably do not function via translation, since they do not overlap significantly with potential ORFs. Large non-coding RNAs are implicated in gene regulation in eukaryotes [100]. Especially RNA2.7 was transcribed massively, making up almost half of the viral transcripts. RNA2.7 inhibits apoptosis by regulation of mitochondria-induced cell death [101]. Furthermore, antisense transcripts were found throughout the genome, but are generally present at a lower level than their sense counterparts. Antisense transcription is increasingly being recognized as being involved in gene regulation, both in pro- and eukaryotes (reviewed in [102]). It can be hypothesized that the antisense transcripts of HCMV provide the virus with an additional mechanism of regulating its expression. Because of the high levels of transcription from non-coding and antisense regions, coding regions only made up one third of transcription. Within these coding regions, splicing patterns were found to be more numerous and complex than previously appreciated. While some of these splicing events are essential for proper expression, others probably have more subtle regulatory roles or could be non-functional by-products of normal transcription. Four new HCMV genes were identified based on this transcriptome study (RL8A, RL9A, UL150A, and US33A; Figure 2), indicating the added value of this kind of study for genome annotation.

The added complexity that is caused by transcription and translation was further illustrated by a study that made use of a novel technique, i.e., ribosome profiling [85]. This technique characterizes the “translatome” by generating libraries of ribosome-protected mRNA fragments. Examining these ribosome footprints, the authors identified 751 ORFs, only 147 of which were previously recognized. ORFs were positioned within existing ORFs (both in-frame and out of frame), upstream of existing ORFs, antisense of existing ORFs, and within presumably non-coding regions. Multiple translation products were also identified on the long non-coding RNAs RNA2.7, RNA1.2, and RNA4.9. Tandem mass spectrometry and protein tagging experiments confirmed the translation of a large proportion of these novel ORFs. Furthermore, this study highlighted the use of alternative 5’ ends, enabling the virus to express different proteins from overlapping coding regions in a temporally regulated fashion.

The discovery that microRNAs (miRNAs), small RNAs that target and silence complementary mRNAs, are not restricted to eukaryotes, but also present in several viruses, including HCMV, added yet another weapon to the viral armory [103,104,105,106,107]. While miRNAs are non-immunogenic and only require minimal space in the genome, they offer the virus an efficient way of regulating both viral and host gene expression. Two studies have used NGS technology to characterize the profile of small RNAs that are expressed in HCMV-infected cells [81,84]. These studies used different HCMV strains (Towne *vs.* AD169), harvested RNA at different time points post-infection (72 h *vs.* 96 h), and assigned and verified novel miRNAs in different ways. The fraction of small RNAs encoding viral miRNAs was 20% and 5%, respectively. The seven miRNAs with the highest expression levels were identical in both, although in a different order. While the second study identified more novel miRNAs from NGS data, the functionality of the only two novel miRNAs reported in the first study was confirmed in transfection assays. The first study also reported that all 22 characterized miRNAs were incorporated into the endogenous host silencing machinery, further highlighting their functionality [81]. Additionally, the authors identified novel small viral RNAs, distinct from miRNAs that were especially observed across the long non-coding RNAs, such as RNA2.7. These could be related to the ribosome footprints that were found on these RNAs [85].

Recently, the HCMV transcriptome of CD14+ and CD34+ cells in experimental and natural latency was characterized through deep sequencing, identifying transcripts that had not previously been related to latency [86]. These include UL44 and UL84 transcripts, normally involved in lytic DNA replication, and the large non-coding RNAs RNA2.7 and RNA4.9.

In addition to providing insights into the viral expression profile during productive and latent infection, NGS-based transcriptome studies can also study the virus-host interface by simultaneously monitoring the changes in cellular transcription. Two studies have already illustrated this for murine cytomegalovirus, but similar studies on HCMV have not yet been published [108,109].

It has to be noted that the complex set of transcripts that were identified through deep sequencing still await further characterization to identify their potential functions. As the authors admit, some of the predicted RNAs and proteins could be aberrant byproducts of normal transcription/translation without further function and/or could be rapidly degraded [82,85]. These studies do offer the first glance at a complex regulatory network that the virus can use to finely balance its replication, including alternative splicing, antisense transcription, large and small non-coding RNAs, and miRNAs.

## 6. Conclusions

A quarter of a century has now passed since the publication of the first complete HCMV genome sequence ushered in the age of HCMV genomics. In the meantime, insights have accumulated regarding the genetic alterations of laboratory-adapted strains, the genome annotation has been progressively fine-tuned, and regions of high nucleotide diversity have been identified. The advent of NGS technology has changed the scope and pace of genomic research and is beginning to show its promise in the HCMV field. However, NGS is still a relatively recent technology and the interpretation of the massive amounts of sequence data requires considerable bioinformatics expertise. Error identification and correction protocols are not completely standardized yet and this precludes the routine application in clinical settings.

Sequencing the complete genome of a clinical HCMV isolate is now possible in a considerably higher throughput and speed than a few years ago. Analysis of a large set of genomes from a diverse group of patients will improve our understanding of the circulating genetic diversity and variability in coding capacity of wild-type HCMV. This could lead to novel insights into the association of genetic diversity and viral pathogenicity, which has eluded the field for years. Furthermore, routine sequencing of transgenic strains to identify unintended genetic alterations should now be possible.

Deep sequencing has shown that intrahost populations of HCMV are remarkably diverse. A better understanding of the dynamics of these populations could have important implications for treatment and vaccine development. The sensitivity of NGS will also improve the standard of drug resistance mutation detection.

RNA-Seq applications have already altered our understanding of the transcriptional complexity during HCMV-infection. Further characterization of these transcripts is warranted and could provide novel insights into mechanisms of viral pathogenicity and potential treatment options. Additionally, simultaneous characterization of the changes in the host transcriptome during infection will reveal currently unknown virus-host interactions.

Meanwhile, a new generation of single-molecule sequencing technologies are being developed [110] or have already found their way to the market [111] (reviewed in [112]). These technologies require much less input material than NGS, making them more attractive for sequencing directly from clinical material. They do not need a library amplification step, ruling out possible artifacts caused by this step. Furthermore, they provide considerable longer read lengths, which facilitate genome assembly and could provide insights into the mutual relations of single variants in intrahost populations. Finally, these technologies can characterize DNA modifications, enabling the direct analysis of epigenetic changes in the genome [113,114]. Undoubtedly, application of single-molecule sequencing to HCMV genomics will aid in deciphering this complex herpesvirus.
